# Growth response of kale (*Brassica oleracea*) and Nile tilapia (*Oreochromis niloticus*) under saline aqua-sandponics-vegeculture system

**DOI:** 10.1038/s41598-023-29509-9

**Published:** 2023-02-10

**Authors:** Fahad Kimera, Muziri Mugwanya, Mahmoud Dawood, Hani Sewilam

**Affiliations:** 1 School of Science and Engineering, Center for Applied Research on the Environment and Sustainability (CARES), The American University in Cairo, AUC Avenue, P.O. Box 74, New Cairo, 11835 Egypt; 2 Department of Animal Production, Faculty of Agriculture, Kafrelsheikh University, Kafrelsheikh, 33516 Egypt; 3 UNESCO Chair in Hydrological Changes and Water Resources Management, RWTH Aachen University, Aachen, Germany

**Keywords:** Plant sciences, Environmental sciences

## Abstract

Salinity and freshwater scarcity are significant challenges affecting agriculture production worldwide. Sustaining food production in arid and semi-arid regions requires innovative, efficient, and low-cost technologies. Integrated aqua-vegeculture systems (IAVS) are promising technologies for cultivating vegetable crops and rearing fish and in a closed-loop system. The system utilizes fish effluents as crop fertilizers and recycles water for increased productivity. Hence, the current study aimed to investigate the response and productivity of kale (*Brassica oleracea* L.) grown at different brackish water salinities in an IAVS. The greenhouse experiment followed a completely randomized design with three salinity variants (i.e., 3000, 6000, and 9000 ppm) and control (freshwater, 400 ppm) with four replicates per treatment. The study results indicated that kale grown in a greenhouse could tolerate salinity levels of up to 6000 ppm without significantly compromising the plants’ growth, yield, and nutritional composition of leaves. Likewise, rearing *Oreochromis niloticus* at high water salinities did not negatively impact the water quality and the growth performance, survival, and feed utilization of fish. Overall, cultivating kale and rearing *O. niloticus* in IAVS in water salinities reaching up to 6000 ppm could be a sustainable agricultural strategy to increase food production in regions affected by freshwater scarcity.

## Introduction

Salinity is one of the most severe abiotic factors affecting crop productivity. It suppresses plant growth, development, and survival by restricting the activation of photosynthesis, protein synthesis, energy, and lipid metabolism due to ionic toxicity, osmotic stress, and oxidative stress arising from saline water in the plant root zone^1^. It is increasingly becoming a serious issue affecting agricultural land productivity worldwide. Approximately 20% of irrigated land (7% of total global land) is affected by salts and is estimated to rise to 50% in the near future^2^. Several direct and indirect factors account for the increasing salinity levels, including climate change, unsustainable use of brackish water, low-quality irrigation water, intensive farming, poor drainage, seawater intrusion, and drought^3^.

Crop growth and establishment vary significantly in relation to salinity tolerance. Osmotic stress and ionic imbalance can affect plant growth in several ways, such as ion toxicity, nutritional disorders, alteration of metabolic processes, membrane disorganization, reduction of cell division and expansion, and DNA damage^4^. Excess salts mostly sodium and chloride ions in the plant tissues, also hinders water and mineral translocation affecting the plant’s enzyme activity, reducing energy production, and other physiological changes^5,6^. Utilization and adoption of low-quality irrigation water are key to sustaining the agricultural industry, mainly in semi- and arid areas. Using saline water in agricultural production causes salinization and reduces crop productivity. However, high-quality fresh water for agriculture becomes less important in areas with high water scarcity levels. Sustaining production in water scarcity areas requires innovative, efficient, low-cost means to manage soil salinity in root zone^7^.

Climate-smart greenhouse farming techniques such as aquaponics and sandponics are among the most efficient solutions for saline agriculture. Such systems are known to be water efficient, easily monitored and controlled, and associated with increased crop yields. Sandponics (SP), also sometimes called Integrated Aqua-Vegeculture system (IAVS) is a growing technique for cultivating plants that utilize sand as a primary medium for mechanical filtration biofilter and as well as growing media for crops. It is a promising sustainable production option for several crops, including vegetables, vines, and fruits^8^.

Sandponics possess greater benefits than other controlled farming systems since sand is readily available in most areas, can be easily sterilized, versatile, easily recycled, and cheaper than soil. This makes SP a more efficient, affordable, and low-risk farming technique in saline zones^9^. The system is tailored to enhance productivity by enabling year-round crop organic production within a controlled environment. Relying solely on such farming systems to solve food security may not be sufficient. However, SP can produce healthy local crops to support a healthy urban/peri-urban lifestyle and eventually help in taking a giant leap toward more nutritious and more food secure communities^10^. The nutrient composition of water effluent in SP plays a major role in the system’s overall performance. The fish wastes contain nitrogen which is presented mainly as total ammonia. Total ammonia consists of both ammonium (NH_4_^+^) and ammonia (NH3); both undergo the nitrification process to oxidize to nitrate (NO_3_−) Nitrogen^11^.

To ensure adequate food security and nutritional balance to face the increasing human population, there is a need to adapt and grow salt-tolerant crops such as vegetables which have a fundamental importance in the human diet due to their nutritional value. Vegetables are essential for human nutrition; however, high salinity levels, especially sodium chloride and sulfates, impact their morphological, physiological, and biochemical functions. They are adequate sources of essential elements, such as fibers and vitamins, carbohydrates, and proteins. Because of the increasing population, the demand for vegetable production is also increasing, which presses the necessity for the increase in their production. Cultivating vegetables, especially in arid areas, requires large amounts of water and fertilizers. Vegetables have been categorized into three groups with respect to their salinity tolerance, i.e., less tolerant such as radish, beans, potato, peas, and sweet pepper; Moderately tolerant such as tomatoes, cucumber, cabbage, onions, watermelon, and broccoli; highly tolerant include beet, kale, turnips, asparagus and gourds^12^.

Kale (*Brassica oleracea* L*.*) is one of the most important high-value vegetables with huge nutritional benefits plus antioxidant activity. It is considered a moderate to highly tolerant vegetable crop to salinity stress^12^. The crop has recorded an increasing consumption rate as a salad mix and salad juice due to its high benefits towards boosting human immunity most, especially in the Covid-19 pandemic^13^. Current literature on morphological, physiological and biochemical responses of kale grown under saline sandponics systems is very limited^1,14,15^. This study aims to investigate the response and productivity of kale grown at different brackish water salinities in an integrated aquaculture agriculture system (sandponics).

## Materials and methods

### Study site

The study was conducted at the Center for Applied Research on the Environment and Sustainability (CARES) at The American University in Cairo, New Cairo, Egypt (30° 01′ 11.7′′ N 31° 29′ 59.8′′ E) from 20th March 2021 to 8th July 2021. The experiment was carried out in a semi-automated fan and pad greenhouse-controlled environment with temperatures ranging from 23 to 25 °C and relative humidity between 60 and 70% during the growing period.

### Experimental design

**Figure 1:**
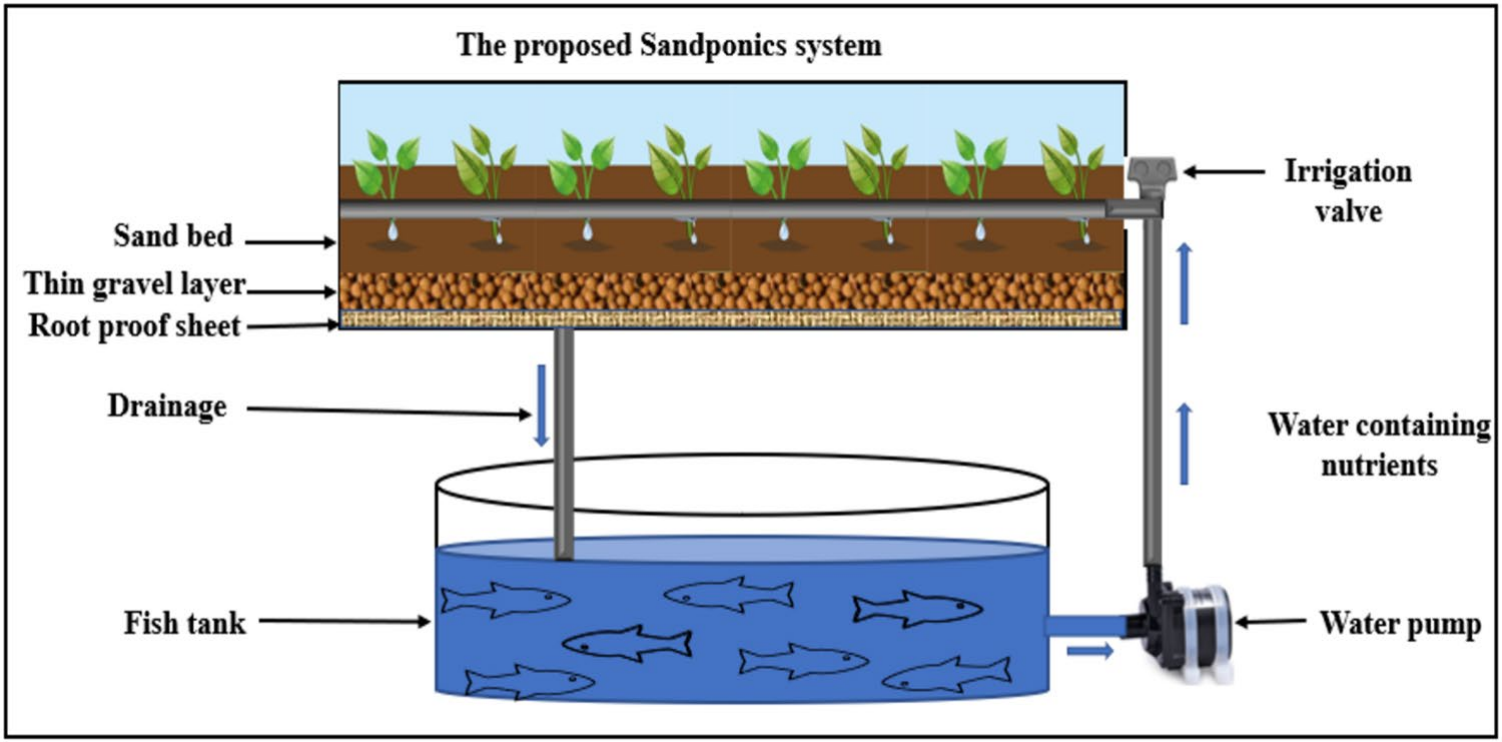
Schematic design of the experimental layout.

The investigation was conducted using a sandponics system and sand as a growth media from a local sand source that had been studied and tested in one of our previous studies^8^. The system model comprised of a plant grow bed filled with sand measuring 30 cm in depth, a fish water tank containing tilapia fish (*Oreochromis niloticus*) stocked at the size of fingerlings stage with 70 L water volume, and a drainage system from the grow beds back to the fish tanks as shown in Fig. 1.

The study followed a completely randomized design with four salinity variants, i.e., a sandponics system operated with normal fresh water with a salinity 400 ppm as the control system (T1), then brackish fish water salinity of 3000 ppm (T2), also brackish fish water salinity 6000 ppm (T3), and lastly, the fourth system was operated water salinity at 9000 ppm (T4). The chemical properties of the salt used in this study are presented in Table 1.

**Table 1:** Chemical properties of the salt used in the study for making different saline waters.

Elements in the dry salt sample			
Sodium chloride	98.5%	Bicarbonate	4 × 10^–3^%
Moisture	0.23%	Iron	3 × 10^–6^%
Insoluble matter	0.02%	Copper	2 × 10^–6^%
Soluble matters	0.57%	Arsenate	2 × 10^–5^%
Calcium	0.0721%	Lead	2 × 10^–5^%
Magnesium	0.0722%	Mercury	5 × 10^–6^%
Sulphate	0.313%	KIO_3_	5.3 × 10^–3^%
Potassium	0.02%	Cadmium	8 × 10^–7^%

All four sandponics systems were designed with the same standard parameters with one exceptional variable i.e., the salinity level of the brackish water in the fish tanks. Each system was composed of four replicates of raised grow beds. The grow beds were designed, customized, and manufactured locally using fiberglass material.

Fish effluent water was pumped to the plant grow bed using a multi-function submersible pump HQB-3500 with a capacity of 50 L/min, 85W, and with a maximum head of 3.5 m supplied from Sensen group of companies, Zhejiang Province, China. Irrigation to the crops was through irrigation drip lines using diaghram emitters connected with valves to ensure uniformity and homogeneity of water applied to each grow bed. Manual emitters were installed at a 30 cm distance from each other. Seedlings were transplanted 5 cm away from the emitters at 30 cm between rows and 30 cm within the row to allow water to flow into the furrows.

All the fish tanks were installed with the same fish stock size of 20 Nile tilapia (*Oreochromis niloticus*) with an average weight of 25 g. The fish was sourced from an already existing fish stock tank at the research center. The fish were fed 3–4 times daily with commercial pellets containing 30% proteins, 5% crude lipid, 6% crude fiber, 13% Ash, and 9% moisture content supplied by Skretting company, Cairo, Egypt. The feeding pattern and frequency were according to the fish body biomass percentage of 2–3% depending on the growth stage and upon reaching satiation.

To maintain the water quality, two full cycles of water recirculation were run every day in batches, and this enabled efficient drainage of water from the sand after each irrigation. Plants were cut upon reaching maturity for at three different time points. Measurable crop parameters included plant height, leaf area, number of leaves per plant, chlorophyll content, fresh weight per plant, dry weights, and nutrient composition. The fish biomass was also monitored on a bi-weekly basis to monitor their relative growth in terms of weight gained and cutting time.

### Plant materials and cultivation practice

Kale seeds–Winterbor variety (*Brassica oleracea*) were imported from Johnny’s selected seeds company in the USA. Seeds were sown in ¼ inch holes in a seed starting mix containing perlite and vermiculite and irrigated with a hand mist sprayer daily to keep the growing media always moist. Sowing was done on 20th March 2021, and seedlings were transplanted when they were four weeks old into raised grow beds.

### Measurement of crop parameters

Kale leaves were cut with a disinfected small cutting scissor, and agronomical trait measurements were taken as an average of a total representative sample of 20 plants per system (Each system had four replicate beds where 5 randomly selected and fixed plants were sampled from each bed/replicate throughout the whole growth cycle.) at 28, 56, and 70 days after transplanting (DAT). Plant heights were taken using a foot ruler and averages were determined. Leaf number was obtained as the number of leaves counted per plant, and averages determined. Leaf length, width, and area were measured using a leaf area meter model CI-202 Portable Laser Leaf Area Meter from CID Bio Science, Washington, USA. Chlorophyll content was measured in the early morning at each cutting time point using MC-100 chlorophyll meter from Apogee Instruments, Inc, Utah, USA, and data was expressed as SPAD averages. Fresh weight was measured using a digital weighing balance, and data was expressed as g/plant.

### Sand test

Sand samples were obtained and sent for analysis at the Soils, Water and Environment Research Institute, Agricultural Research Center, Giza, Egypt. The soil particle size distribution was carried out using the pipette method. Electrical conductivity (EC) values were measured from the soil paste extract; soil pH values were taken from soil suspensions at a ratio of 1:2.5, as described by Estefan^16^. The available nitrogen in the soil sample was extracted using potassium chloride (KCl) as an extractable solution with the ratio of (5gm soil to 50 mL KCl) and determined using the micro-kjeldahl method. Available potassium was determined using a flame photometer, and the other elements in the soil sample were determined by using inductively coupled plasma (ICP) Spectrometry (model Ultima 2 JY Plasma). The physical and chemical characteristics of the used sand at the start of the experiment are presented in Table 2.

**Table 2:** The physical and chemical properties of the experimented sand.

pH 1:2.5	EC ppm	SP	Macro and micronutrients (mg/kg)					Anions and cations (meq./L)				
			N	P	K	Mn	Fe	Cl^−^	SO4^–^	Ca^++^	HCO_3_	Mg^++^
7.9	2355.2	18	53	0.02	29	0.19	1.20	16.10	22.40	23.60	1.89	10

### Water quality analysis and fish growth performance

Fish water quality parameters such as water temperature, pH, and dissolved oxygen (DO) were closely monitored using automated digital Nilebot technologies by Conative labs to adjust to the ideal required levels as reported by Somerville et al.^17^. In contrast, ammonia, nitrite, and nitrate were monitored using an API test kit twice every week. However, water quality lab analysis was performed once a week to record respective water quality data. Briefly, 100 mL of freshwater samples were collected from the fish tanks before feeding and immediately taken to the lab for detection of ammonia, nitrate and nitrite concentrations using a photometer along with the ammonia reagent kit (H193715-01), nitrate reagent kit (H193728-01), and nitrite reagent kit (H193707-01) respectively from HANNA instruments. Specific absorbance of the nitrogenous elements was measured using the Aquaculture Photometer device (H183303). The device was set to display the concentrations of ammonia–nitrogen, ammonium, nitrate–nitrogen, and nitrite-nitrogen in mg/L. Real-time analysis results were presented in the manuscript as mean values read from the photometer.

Fish growth parameters such as feed conversion ratio (FCR), specific growth rate (SGR), feed intake (FI), body weight gain (BWG), and survival rate (SR%) were calculated according to the formula below.

- BWG = Final body weight − Initial body weight.
- FCR = FI/BWG.
- SGR = (ln (Final body weight) − ln (Initial body weight))/Number of days.
- SR = (Number of fish at the end of the study/Number of fish at the beginning of the experiment) × 100.

### Nutrient composition analysis of leaves

According to Official methods of analysis from the association of official analytical chemists (A.O.A.C 1990)^18^ moisture content and Vitamin C were determined. Vitamin A was determined according to the procedures described by Aremu and Nweze^19^. Briefly, 100 g of the sample were homogenized, from which 1 g was obtained and soaked in 5 mL methanol for two hours at room temperature in the dark for complete extraction of a pro-vitamin A carotenoid, β-carotene. Separation of the β-carotene layer was achieved through the addition of hexane to the sample, and moisture was removed using sodium sulphonate. The absorbance of the layer was measured at 436 nm using hexane as a blank. β-carotene was calculated using the formula:

$$\upbeta {\text{-carotene }}\left( {\mu {\text{g}}/{1}00{\text{ g}}} \right) \, = {\text{ Absorbance }}\left( {\text{436 nm}} \right) \, \times {\text{ V }} \times {\text{ D }} \times { 1}00 \, \times { 1}00/{\text{W }} \times {\text{ Y,}}$$

where V = total volume of the extract; *D* Dilution factor; *W* Sample weight; *Y* Percentage dry matter content of the sample.

Vitamin A was then determined according to the concept of Retinol Equivalent (RE) of the β-carotene content of the vegetables using the standard conversion formula. Total hydrolyzable carbohydrates were determined as glucose using phenol–sulfuric acid reagent as described by Michel^20^. For the determination of protein and mineral content, 0.5 g of dried samples were digested using sulfuric acid (H2SO4) and hydrogen peroxide (H2O2) as described by Cottenie^21^. From the extracted sample, the following minerals were determined:

Nitrogen was determined according to the procedures described by Plummer^22^. Briefly, 5 mL of the digestive solution was distilled with 10 mL of sodium hydroxide (NaOH) for 10 min to obtain ammonia. Back titration was then used to determine the amount of nitrogen present in ammonia. Protein content was calculated by multiplying total nitrogen by 6.25.

Phosphorus content was determined calorimetrically (660 nm) according to the procedures described by Jackson^23^. Potassium, Calcium, and Sodium were determined against a standard using a flame-photometer (JEN way flame photometer) as described by Piper^24^. Magnesium (Mg), Copper (Cu), Manganese (Mn), Zinc (Zn), and Iron (Fe) content were determined using Atomic Absorption Spectrophotometer, Pyeunican SP1900, according to methods described by Bradfield and Spincer^25^.

### Statistical analysis

All data collected were analyzed using IBM-SPSS statistical tool (Version 22) and expressed as Mean ± SD. These data were subjected to a Leven’s test before analysis of variance (ANOVA) was conducted. ANOVA (one-way and two-way ANOVA) was performed to detect significant differences in all the measured parameters. The difference in means was analyzed by Duncan multiple range test (DMT) at *α* = 0.05.

### Plant material

All plant materials and related procedures in this study were done in accordance with the guidelines of the Institutional Review Board of the American University in Cairo and the Ministry of Agriculture and Land Reclamation in Egypt.

### Ethics approval

This study followed the guidelines and approval of Committee of Animal Welfare and Research Ethics, Faculty of Agriculture, Kafrelsheikh University, Egypt.

## Results

### Water quality parameters

The water quality parameters analyzed during the experimental period (Table 3) showed that the average water temperature values ranged between 24.30 and 24.90 °C with no marked variation among the treatments. The dissolved oxygen concentration (DO) was higher in control than in other treatments, with the lowest values recorded in the 9000 ppm. The average ammonium (NH_4_^+^) concentrations varied across treatments, with the control and 6000 ppm recording the lowest and highest values, respectively. Similarly, the average ammonia (NH_3_) and ammonia–nitrogen (NH_3_–N) concentrations varied across treatments, with the control and 6000 ppm recording the lowest and highest values, respectively. The average nitrate (NO_3_^_^) concentrations were higher in 3000 ppm compared to other treatments, with 6000 ppm recording the lowest values. The concentrations of nitrate-nitrogen (NO_3−_–N) were lower in 3000 ppm and 6000 ppm compared to other treatments as shown in Table 3. Data on the nitrite (NO_2_^_^) and nitrite-nitrogen (NO_2−_–N) concentrations indicated that the 6000 ppm salinity treatment had the highest levels compared to other treatments.

**Table 3:** Average water quality values of freshwater and saline water used for rearing Nile tilapia (*Oreochromis niloticus*) during the study period*.*

Water-quality parameters	Control	3000 ppm	6000 ppm	9000 ppm
Temp (°C)	24.70	24.60	24.30	24.90
DO (mg/L)	7.31	5.63	6.06	5.27
NH_4_^+^ (mg/L)	1.44	2.23	4.39	2.42
NH_3_ (mg/L)	1.35	2.10	2.36	2.28
NH_3_–N (mg/L)	1.14	1.73	1.89	1.87
NO_3_^_^ (mg/L)	83.00	99.68	59.41	89.37
NO_3_^_^–N (mg/L)	18.74	10.28	13.42	20.69
NO_2_^_^ (mg/L)	18.90	16.20	20.10	19.60
NO_2_^_^–N (mg/L)	5.80	5.10	6.10	6.00

Water quality parameters, *Temp* temperature, *DO* dissolved oxygen, *NH*_*4*_^+^ Ammonium, *NH*_*3*_ Ammonia, *NH*_*3*_*-N* Ammonia–nitrogen, *NO*_*3*_^*_*^ nitrates, *NO*_*3*_^*_*^*–N* nitrate-nitrogen, *NO*_*2*_^*_*^ nitrites, *NO*_*2*_^*_*^*–N* nitrite-nitrogen.

### Fish performance

Data on the growth performance and feed utilization of fish (Table 4) showed no significant differences were noted in the final body weight, body weight gain, feed conversion ratio, specific growth rate and survival among all the treatments.

**Table 4:** Growth performance of Nile tilapia (*Oreochromis niloticus*) reared in sandponics systems during the study period.

	Control	3000 ppm	6000 ppm	9000 ppm
IBW (g)	31.75 ± 0.29^b^	31.63 ± 0.85^b^	32.00 ± 0.41^b^	33.00 ± 0.71^a^
FBW (g)	73.18 ± 10.18^a^	66.10 ± 10.34^a^	69.68 ± 12.56^a^	65.45 ± 14.43^a^
FI (g)	1791.58	1716.95	1710.92	1816.25
BWG (g)	829.75 ± 201.63^a^	689.50 ± 206.79^a^	753.50 ± 251.20^a^	648.50 ± 288.53^a^
FCR	2.26 ± 0.58^a^	2.73 ± 1.06^a^	2.48 ± 0.82^a^	3.24 ± 1.39^a^
SGR (%)	1.18 ± 0.19^a^	1.04 ± 0.25^a^	1.09 ± 0.26^a^	0.95 ± 0.28^a^
Survival rate (%)	100 ± 0.00^a^	100 ± 0.00^a^	100 ± 0.00^a^	100 ± 0.00^a^

Data represented as mean ± SD (n = 3). Means in the same row having different lower superscript letters are significantly different at *p* < 0.05.

*IBW* initial body weight, *FBW* final body weight, *FI* feed intake, *BWG* body weight gain, *FCR* feed conversion ratio, *SGR* specific growth rate.

### Agronomical parameters

The effect of different treatments on plant height, leaf number, leaf area, and SPAD at different time points are presented in Table 5. At Cuts 1, 2, and 3, data on plant height showed that the control treatment significantly recorded the highest values (*p* < 0.05) compared to other treatments. However, the plant heights for 3000 ppm treatment increased by 5.3% and 21.2% at Cuts 2 and 3, respectively, whereas a decline in plant height was noted in 6000 and 9000 ppm treatments, especially at Cut 2. There was a significant interaction (*p* < 0.0001) between treatments x DAT.

**Table 5:** Plant height, leaf number, leaf area and SPAD values of kale cultivated under freshwater and different salinities (3000 ppm, 6000 ppm, and 9000 ppm) at different cuts.

Agronomical traits	Cut number			Average
	Cut 1	Cut 2	Cut 3	
Plant height (cm)				
Control	24.40 ± 3.35^a^	26.15 ± 2.70^a^	30.60 ± 2.70^a^	27.05 ± 3.20^a^
3000 ppm	20.55 ± 2.41^b^	21.63 ± 2.74^b^	24.90 ± 3.66^b^	22.36 ± 2.27^b^
6000 ppm	20.73 ± 3.87^b^	19.53 ± 3.56^c^	19.45 ± 2.62^c^	19.90 ± 0.72^bc^
9000 ppm	16.98 ± 2.34^c^	16.93 ± 1.85^d^	16.98 ± 1.87^d^	16.96 ± 0.03^c^
Leaf number (Plant^−1^)				
Control	14.10 ± 1.07^a^	6.75 ± 0.64^a^	7.50 ± 1.15^a^	9.45 ± 4.04^a^
3000 ppm	14.20 ± 1.47^a^	6.20 ± 0.62^b^	7.25 ± 1.29^b^	9.22 ± 4.35^a^
6000 ppm	13.75 ± 1.12^a^	5.80 ± 0.62^b^	5.30 ± 0.86^c^	8.28 ± 4.74^a^
9000 ppm	12.95 ± 1.00^b^	4.90 ± 0.92^c^	4.10 ± 1.02^d^	7.32 ± 4.89^a^
Leaf area (cm^2^)				
Control	147.20 ± 10.78^a^	184.07 ± 22.33^a^	220.23 ± 23.08^a^	183.83 ± 36.52^a^
3000 ppm	114.38 ± 12.71^b^	155.34 ± 21.69^b^	166.30 ± 21.14^b^	145.34 ± 27.37^a^
6000 ppm	98.38 ± 15.69^c^	147.06 ± 15.34^b^	136.68 ± 28.29^c^	127.37 ± 25.64^a^
9000 ppm	92.04 ± 14.41^c^	141.69 ± 9.90^b^	124.38 ± 26.75^c^	119.37 ± 25.20^a^
SPAD				
Control	38.96 ± 2.91^c^	22.48 ± 6.06^b^	27.40 ± 7.28^b^	29.61 ± 8.46^a^
3000 ppm	45.66 ± 3.36^b^	35.36 ± 11.84^a^	32.66 ± 7.22^a^	37.89 ± 6.87^a^
6000 ppm	48.95 ± 4.18^a^	32.57 ± 7.89^a^	26.63 ± 5.05^b^	36.05 ± 11.59^a^
9000 ppm	46.30 ± 4.62^b^	34.14 ± 8.20^a^	30.41 ± 4.75^ab^	36.95 ± 8.31^a^

Data expressed as mean ± SD (n = 5). Means in the same column at the same cutting time point having different lower superscript letters are significantly different at *p* < 0.05.

Results on leaf number per plant indicated a decline in leaf number across all treatments at different Cuts with 9000 ppm significantly recording the lowest leaf number per plant at Cuts 1, 2, and 3 compared to other treatments (*p* < 0.05). There was a significant interaction (*p* < 0.0001) between treatments × DAT.

For leaf area, the control treatment exhibited an increase in the percentage leaf area by 25.1% and 49.6% at Cuts 2 and 3, respectively. Likewise, 3000 ppm treatment recorded an increase in leaf area percentage by 35.8% and 45.4% at Cuts 2 and 3, respectively. However, 6000 ppm and 9000 ppm recorded a decline in the percentage leaf area by 7.1% and 12.2% at Cut 3, respectively. Overall, the control treatment significantly had the highest leaf area (*p* < 0.05) compared to other treatments. A significant interaction (*p* < 0.0001) between treatments × DAT was noted.

Results on SPAD indicate a variation in SPAD values across all treatments at different Cuts. Generally, the average SPAD values for all treatments decreased at Cuts 2 and 3, with the control significantly recording the lowest SPAD values (*p* < 0.05) compared to other treatments. There was a significant interaction (*p* < 0.0001) between treatments × DAT.

### Herbage yield

**Figure 2:**
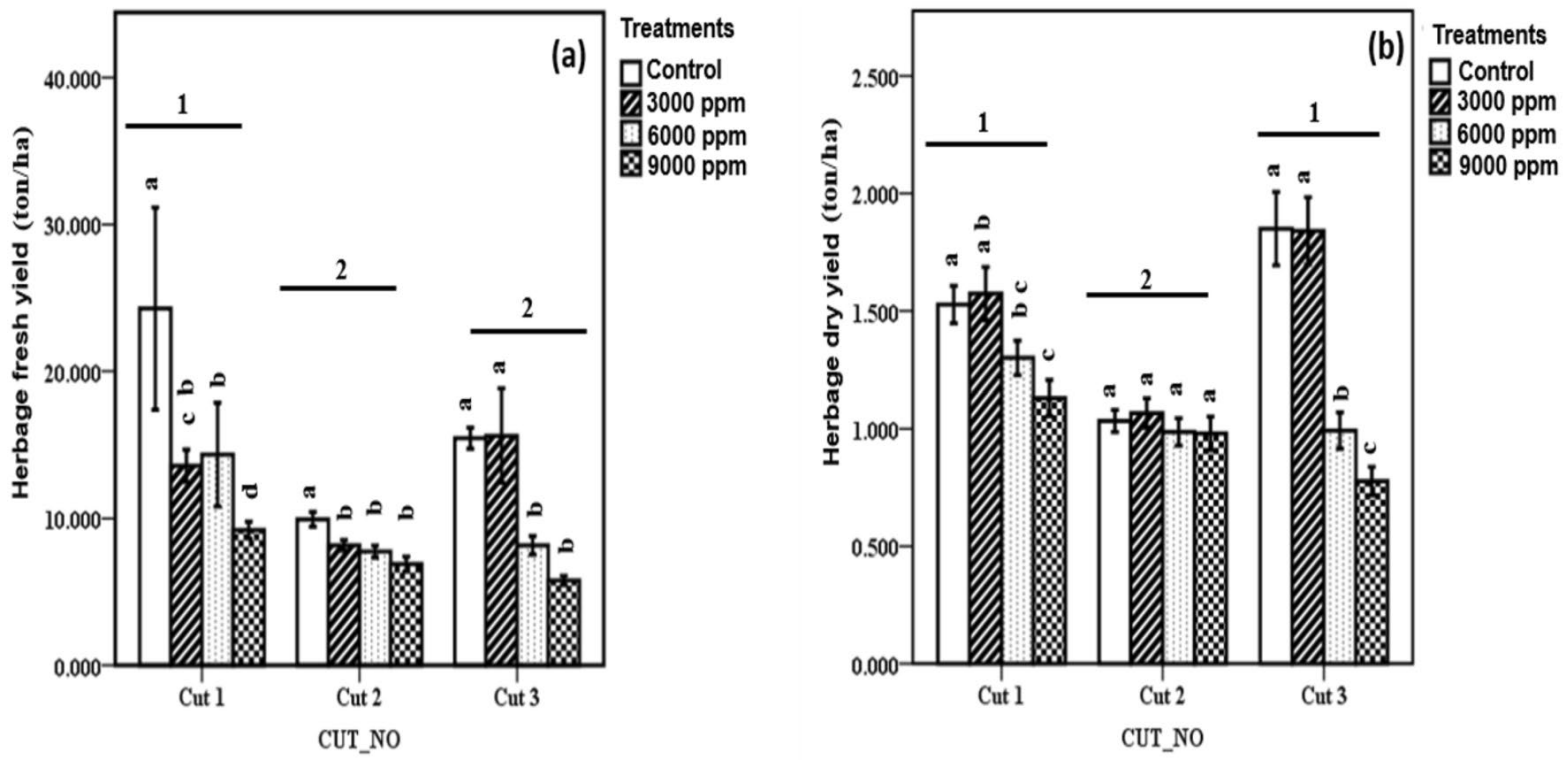
(**a**) Herbage fresh yield and (**b**) herbage dry yield of kale cultivated under fresh water (control) and different salinities (3000 ppm, 6000 ppm, and 9000 ppm) at different cutting time points. Data expressed as mean ± SE. Error bars represent the standard error. Bar columns within the same cutting time point having different letters are significantly different (*p* < 0.05). Horizontal bars at the top of bar columns having different numbers indicate a significant difference between cutting time points (*p* < 0.05).

Results on the effect of different water salinities on the herbage fresh and dry yield of kale (Fig. 2) were presented at different cutting time points. As shown in Fig. 2A, data on fresh yield in cut 1 indicated that the control significantly had the highest herbage yield (*p* < 0.05) (24. 28 ton/ha) compared to 3000 ppm (13.58 ton/ha), 6000 ppm (14.35 ton/ha), and 9000 ppm (9.21 ton/ha). Likewise, in cut 2, the control significantly recorded the highest fresh herbage yield (9.94 ton/ha) (*p* < 0.05) compared to 3000 ppm (8.17 ton/ha), 6000 ppm (7.75 ton/ha), and 9000 ppm (6.89 ton/ha). In cut 3, results indicated that the control and 3000 ppm salinity treatment significantly recorded higher values for fresh herbage yield (15.47 and 15.62 ton/ha respectively) (*p* < 0.05) compared to 6000 ppm (8.17 ton/ha) and 9000 ppm (5.77 ton/ha). Overall, cut 1 significantly had the highest average fresh yield compared to cut 2 and cut 3 (*p* < 0.05).

Results on the herbage dry yield of kale are presented in Fig. 2B. In cut 1, plants cultivated in the freshwater sandponics (control) and 3000 ppm significantly recorded a higher herbage dry yield (1.53 and 1.57 ton/ha respectively) (*p* < 0.05) compared to 6000 and 9000 ppm (1.30 and 1.13 ton/ha respectively). However, in cut 2, the dry herbage yield declined by 32.7, 31.9, 23.9, and 13.3% in control, 3000, 6000, and 9000 ppm, respectively. However, no significant difference in the dry herbage yield was not among all the treatments. In cut 3, the control and 3000 ppm salinity treatments significantly recorded a percentage increase in their herbage yield by 17.3 and 14.7%, respectively (*p* < 0.05), compared to 6000 and 9000 ppm. Overall, cut 1 and 3 significantly recorded a higher average dry yield than cut 2 (*p* < 0.05).

### Soil physical and compositional changes during the study

Results on the soil physical and composition changes during the study (Table 6) showed no significant differences in soil pH and particle size distribution across all treatments. However, the 9000 ppm salinity treatment significantly recorded higher values for electroconductivity (EC) (*p* < 0.05) compared to other treatments. Data on anions showed no significant differences in the contents of hydrogen carbonate (HCO3−) and sulfate (SO4−) ions among all treatments. However, the 9000 ppm salinity treatment significantly recorded the highest chloride ions (Cl^−^) content (*p* < 0.05) compared to other treatments. Likewise, the 9000 ppm salinity treatment significantly recorded the highest sodium ions (Na^+^), calcium ions (Ca^2+^), and magnesium ions (Mg^2+^) (*p* < 0.05) content compared to other treatments. Results on the macronutrient composition of soil indicated no significant differences in phosphorus (P) and potassium (K) composition among all treatments. However, the nitrogen (N) composition of the soil was significantly lower (*p* < 0.05) in the 3000 ppm salinity treatment compared to other treatments. For microelement composition, no significant differences in manganese (Mn) and zinc (Zn) content were noted across all treatments. However, the iron composition of the soil was significantly lower (*p* < 0.05) in the 6000 and 9000 ppm treatments compared to the 3000 ppm and control. Likewise, the copper (Cu) soil composition was significantly lower (*p* < 0.05) in 6000 ppm salinity treatment compared to 3000 ppm and control.

**Table 6:** The physical and chemical properties of the experimented sand at final harvesting*.*

Treatment	Anions and cations (meq./L)									
	pH	EC (dS/m)	SP	HCO_3_^−^	Cl^−^	SO_4_^−^	Ca^2+^	Mg^2+^	Na^+^	K^+^
Control	8.56 ± 0.04^a^	1.98 ± 0.33^b^	20.00 ± 0.00^a^	0.75 ± 0.35^a^	12.00 ± 3.53^b^	7.03 ± 0.63^a^	6.00 ± 0.71^b^	4.00 ± 0.71^c^	9.27 ± 1.87^b^	0.45 ± 0.04^b^
3000 ppm	8.58 ± 0.08^a^	2.90 ± 0.30^b^	20.00 ± 1.41^a^	1.75 ± 0.35^a^	17.25 ± 2.47^b^	9.92 ± 0.21^a^	9.00 ± 0.71^b^	5.50 ± 0.00^b^	13.95 ± 2.26^b^	0.47 ± 0.07^b^
6000 ppm	8.60 ± 0.04^a^	2.93 ± 0.18^b^	20.50 ± 0.71^a^	1.50 ± 0.00^a^	17.00 ± 2.12^b^	10.73 ± 0.35^a^	9.00 ± 0.71^b^	6.50 ± 0.00^b^	13.18 ± 1.03^b^	0.55 ± 0.04^ab^
9000 ppm	8.59 ± 0.05^a^	4.56 ± 0.90^a^	21.00 ± 0.00^a^	2.00 ± 0.71^a^	28.5 ± 4.24^a^	15.01 ± 4.02^a^	15.00 ± 2.12^a^	9.00 ± 0.71^a^	22.60 ± 3.68^a^	0.67 ± 0.02^a^

Treatment	Macro and micro elements in mg/kg									
	N	P	K	Fe	Cu	Mn	Zn			
Control	119.50 ± 34.65^a^	6.41 ± 0.69^a^	41.50 ± 0.71^a^	0.81 ± 0.03^a^	0.06 ± 0.00^a^	0.28 ± 0.05^a^	0.16 ± 0.01^a^			
3000 ppm	54.00 ± 1.41^b^	6.82 ± 2.12^a^	48.50 ± 9.19^a^	0.81 ± 0.02^a^	0.06 ± 0.01^a^	0.27 ± 0.01^a^	0.19 ± 0.00^a^			
6000 ppm	129.00 ± 15.56^a^	4.91 ± 0.30^a^	57.50 ± 6.36^a^	0.59 ± 0.07^b^	0.04 ± 0.00^b^	0.36 ± 0.02^a^	0.16 ± 0.01^a^			
9000 ppm	102.50 ± 28.99^a^	8.36 ± 2.80^a^	53.00 ± 2.83^a^	0.54 ± 0.13^b^	0.05 ± 0.00^ab^	0.30 ± 0.05^a^	0.19 ± 0.01^a^			

Data expressed as mean ± SD (n = 3).

*EC* electroconductivity, *SP* particle size distribution, *HCO*_*3*_^*−*^ hydrogen carbonate ions, Cl^−^ chloride ions, *SO*_*4*_^*−*^ sulfate ions, *Ca*^*2+*^: calcium ions, *Mg*^*2+*^ magnesium ions, *Na*^*+*^ sodium ions, *K*^*+*^ potassium ions, *N* nitrogen, *P* phosphorus, *K* potassium, *Fe* iron, *Cu* copper, *Mn* manganese, *Zn* zinc.

Different lower superscript letters within the same column indicate a significant difference within the treatments at *p* < 0.05.

### Leaf nutrient composition, free proline, and sodium ion content in leaves

The data on moisture percentage, total carbohydrates, proteins, vitamin A, and vitamin C content (Table 7) indicated no significant differences among all the treatments (Table 7). However, vitamin A content decreased among all treatments with increasing cutting time points. Results on the proline content in leaves indicated no significant differences among all treatments. However, the sodium ion (Na^+^) concentration of leaves varied across the cutting time points, with 3000, 6000, and 9000 ppm having a percentage increase in the Na^+^ concentration in cut 2 (19.5, 10.2, and 6.1%, respectively) and cut 3 (23.9, 33.5, and 18.3% respectively) relative to cut 1.

**Table 7:** Nutrient composition, proline and sodium ion concentration values of kale leaves cultivated under freshwater and different salinities (3000 ppm, 6000 ppm, and 9000 ppm) at different cutting time points.

Treatments	Moisture (%)	Total carbs (g/100 g DW)	Proteins (g/100 g DW)	Vit. A (mg/100 g FW)	Vit. C (mg/100 g FW)	Proline (mg/100 g DW)	Na^+^ (%)
Cut 1							
Control	86.20 ± 0.74^aA^	57.95 ± 3.14^aA^	0.13 ± 0.02^aA^	1.78 ± 0.24^aA^	25.88 ± 4.55^aB^	69.59 ± 20.86^aA^	1.28 ± 1.23^cB^
3000 ppm	85.65 ± 0.87^aA^	54.24 ± 12.34^aA^	0.17 ± 0.01^aA^	1.51 ± 0.10^aA^	25.99 ± 3.17^aB^	84.13 ± 15.86^aA^	1.59 ± 0.11^bB^
6000 ppm	84.91 ± 0.80^aA^	51.53 ± 8.57^aA^	0.15 ± 0.03^aA^	1.37 ± 0.10^aA^	24.92 ± 0.78^aB^	76.47 ± 19.06^aA^	1.67 ± 0.71^bB^
9000 ppm	85.06 ± 1.27^aA^	47.53 ± 11.73^aA^	0.16 ± 0.03^aA^	1.44 ± 0.34^aA^	24.29 ± 2.86^aB^	83.91 ± 58.14^aA^	1.97 ± 0.33^aB^
Cut 2							
Control	87.38 ± 1.32^aA^	200.95 ± 83.46^aA^	0.13 ± 0.01^aA^	0.95 ± 0.35^aB^	28.20 ± 2.78^aA^	64.69 ± 11.04^aA^	1.38 ± 0.99^bAB^
3000 ppm	86.19 ± 0.67^aA^	65.19 ± 3.50^aA^	0.15 ± 0.04^aA^	0.99 ± 0.31^aB^	32.89 ± 5.67^aA^	103.88 ± 43.28^aA^	1.90 ± 0.19^aAB^
6000 ppm	85.24 ± 1.37^aA^	52.72 ± 13.85^aA^	0.15 ± 0.03^aA^	1.11 ± 0.40^aB^	39.93 ± 11.57^aA^	101.07 ± 25.36^aA^	1.84 ± 0.15^aAB^
9000 ppm	84.73 ± 1.86^aA^	53.75 ± 11.46^aA^	0.17 ± 0.02^aA^	0.90 ± 0.25^aB^	29.09 ± 5.76^aA^	61.43 ± 17.32^aA^	2.09 ± 0.36^aAB^
Cut 3							
Control	88.46 ± 0.51^aA^	59.21 ± 2.38^aA^	0.13 ± 0.02^aA^	1.07 ± 0.48^aB^	29.77 ± 1.98^aA^	71.46 ± 9.30^aA^	1.35 ± 0.10^bA^
3000 ppm	86.81 ± 0.79^aA^	62.19 ± 5.61^aA^	0.15 ± 0.04^aA^	1.23 ± 0.40^aB^	29.22 ± 2.21^aA^	75.47 ± 26.64^aA^	1.97 ± 0.30^aA^
6000 ppm	86.72 ± 1.16^aA^	47.35 ± 3.48^bA^	0.16 ± 0.03^aA^	0.63 ± 0.17^aB^	30.35 ± 3.13^aA^	96.40 ± 28.14^aA^	2.23 ± 0.22^aA^
9000 ppm	83.84 ± 2.23^bA^	62.82 ± 3.48^aA^	0.12 ± 0.02^aA^	0.67 ± 0.13^aB^	27.31 ± 2.04^aA^	87.21 ± 41.76^aA^	2.33 ± 0.23^aA^

Data expressed as mean ± SD (n = 3).

Different lower superscript letters within the same column at the same cutting time point indicate a significant difference within the treatments at *p* < 0.05. Different upper superscript letters within the same column indicate a significant difference between the cuts at *p* < 0.05.

As shown in Table 8, no significant differences in the leaf concentrations of macro-elements (nitrogen [N], phosphorus [P], potassium [K], magnesium [Mg], and calcium [Ca]) were noted among all treatments. The P content of leaves in cut 2, however, increased by 92.3 and 18.4% in 6000 ppm and 9000 ppm, respectively. Furthermore, the K content of leaves in cut 2 increased by 11.3, 16.3, 1.5, and 19.8% in control, 3000, 6000, and 9000 ppm, respectively. Data on the Mg content of leaves in cut 2 showed a percentage decrease of 21.7, 15.0, 30.9, and 15.3% in control, 3000, 6000, and 9000 ppm, respectively whereas in cut 3, a percentage decrease of 24.7, 25.4, 29.1, 18.3% was noted in the control, 3000, 6000, and 9000 ppm respectively. For Ca content in cut 2, the results indicated a percentage decrease of 19.0, 57.8, and 25.2% in 3000, 6000, and 9000 ppm, respectively, whereas in cut 3 a percentage decrease of 17.8, 62.7, and 48.5% was noted in 3000, 6000, and 9000 ppm respectively.

**Table 8:** Macro and micro-elements in kale leaves cultivated under freshwater and different salinities (3000 ppm, 6000 ppm, and 9000 ppm) at different cutting time points.

Treatments	N (%)	P (%)	K (%)	Mg (mg/100 g)	Ca (mg/100 g)	Zn (mg/100 g)	Cu (mg/100 g)	Mn (mg/100 g)	Fe (mg/100 g)
Cut 1									
Control	2.35 ± 0.71^aA^	0.37 ± 0.05^aB^	2.75 ± 0.18^aB^	380.26 ± 47.91^aA^	385.03 ± 177.68^aA^	17.25 ± 1.02^aB^	5.34 ± 0.35^aC^	8.86 ± 1.09^aA^	198.89 ± 37.44^aB^
3000 ppm	1.99 ± 0.37^aA^	0.46 ± 0.13^aB^	2.52 ± 0.27^aB^	316.35 ± 50.33^aA^	385.32 ± 226.48^aA^	16.58 ± 2.11^aB^	4.85 ± 0.74^aC^	6.06 ± 1.12^aA^	178.67 ± 44.95^aB^
6000 ppm	1.98 ± 0.37^aA^	0.39 ± 0.04^aB^	2.71 ± 0.16^aB^	328.80 ± 36.99^aA^	657.69 ± 260.41^aA^	16.78 ± 1.98^aB^	3.96 ± 0.49^bC^	8.61 ± 1.84^aA^	163.41 ± 33.15^aB^
9000 ppm	2.17 ± 0.39^aA^	0.38 ± 0.07^aB^	2.53 ± 0.31^aB^	330.01 ± 44.59^aA^	423.97 ± 229.58^aA^	14.85 ± 3.37^aB^	3.91 ± 0.63^bC^	8.45 ± 1.66^aA^	193.60 ± 43.59^aB^
Cut 2									
Control	2.51 ± 0.56^aA^	0.49 ± 0.09^aA^	3.06 ± 0.18^aA^	297.66 ± 32.43^aB^	477.08 ± 208.52^aB^	17.58 ± 1.42^aA^	6.58 ± 0.18^aB^	9.07 ± 1.20^aA^	182.85 ± 25.93^aB^
3000 ppm	2.47 ± 0.21^aA^	0.47 ± 0.08^aA^	2.93 ± 0.13^aA^	268.80 ± 44.28^aB^	312.10 ± 83.67^aB^	18.22 ± 2.06^aA^	7.22 ± 0.73^aB^	5.60 ± 0.78^aA^	184.53 ± 33.72^aB^
6000 ppm	2.36 ± 0.45^aA^	0.75 ± 0.30^aA^	2.75 ± 0.24^aA^	226.90 ± 23.50^aB^	277.62 ± 93.80^aB^	18.60 ± 0.83^aA^	6.25 ± 0.61^aB^	7.89 ± 2.62^aA^	186.23 ± 34.77^aB^
9000 ppm	1.99 ± 0.31^aA^	0.45 ± 0.08^aA^	3.03 ± 0.22^aA^	279.47 ± 33.61^aB^	317.34 ± 101.50^aB^	17.86 ± 1.43^aA^	6.95 ± 0.94^aB^	7.72 ± 1.42^aA^	174.58 ± 22.92^aB^
Cut 3									
Control	2.69 ± 0.38^aA^	0.44 ± 0.07^aB^	2.31 ± 0.27^aC^	286.42 ± 27.52^aB^	338.61 ± 53.16^aB^	18.11 ± 1.13^aA^	7.85 ± 0.51^abA^	7.08 ± 1.73^aA^	226.64 ± 15.57^abA^
3000 ppm	2.81 ± 0.24^aA^	0.41 ± 0.10^aB^	2.24 ± 0.41^aC^	235.95 ± 32.65^aB^	316.61 ± 66.11^aB^	20.16 ± 0.65^aA^	8.69 ± 0.16^aA^	7.47 ± 1.32^aA^	252.50 ± 14.10^aA^
6000 ppm	2.18 ± 0.56^aA^	0.49 ± 0.12^aB^	2.24 ± 0.40^aC^	233.22 ± 14.73^aB^	245.05 ± 61.58^aB^	18.61 ± 1.49^aA^	7.26 ± 0.61^bA^	8.61 ± 2.47^aA^	194.44 ± 41.96^bA^
9000 ppm	1.99 ± 0.79^aA^	0.41 ± 0.06^aB^	2.64 ± 0.31^aC^	269.46 ± 30.39^aB^	218.35 ± 59.51^aB^	18.98 ± 0.77^aA^	8.10 ± 0.70^abA^	8.05 ± 3.46^aA^	224.73 ± 13.69^abA^

Data expressed as mean ± SD (n = 3).

*N* nitrogen, *P* phosphorus, *K* potassium, *Mg* magnesium, *Ca* calcium, *Zn* zinc, *Cu* copper, *Mg* manganese, *Fe* iron.

Different lower superscript letters within the same column at the same cutting time point indicate a significant difference within the treatments at *p* < 0.05. Different upper superscript letters within the same column indicate a significant difference between the cuts at *p* < 0.05.

Results of the micro-element composition (zinc [Zn] and manganese [Mn] indicated no significant differences among all treatments. However, the copper (Cu) content of leaves varied across treatments, with 6000 ppm significantly recording lower values in cut 3 (*p* < 0.05) compared to 3000 ppm. Overall, the Cu content of leaves in cut 2 significantly increased (*p* < 0.05) by 23.2, 48.9, 57.8, and 77.8% in control, 3000, 6000, and 9000 ppm, respectively, whereas in cut 3 it significantly increased (*p* < 0.05) by 47.0, 79.2, 83.3, and 107.2% in control, 3000, 6000, and 9000 ppm respectively. No significant differences in the iron (Fe) content were noted among all treatments in cut1 and cut 2. However, in cut 3, 6000 ppm significantly recorded lower values for Fe content (*p* < 0.05) compared to 3000 ppm. Overall, the Fe content in cut 3 significantly increased (*p* < 0.05) by 13.9, 41.3, 18.9, and 16.1% in control, 3000, 6000, and 9000 ppm, respectively.

## Discussion

There is an inverse relationship between salinity and dissolved oxygen levels (DO)^26^. In this study, the concentrations of DO were lower in saline water treatments compared to the control. However, these concentrations were within the optimum range (5.0 to 7.5 mg/L) for the production of *O. niloticus,* as reported by DeLong et al.^27^. Likewise, in a normally functioning system, the concentrations of NH_3_–N and NO_2−_–N must be below 1 and 5 mg/L respectively for the growth and survival of freshwater fish species^28^. However, in this study, the concentrations of NO_2_^_^–N in the saline experimental groups were slightly above the desirable range, which could be attributed to salinity-associated changes in the microbial diversity and abundance of nitrifying bacteria (*Nitrosomonas* sp. and *Nitrobacter* sp.). Navada et al.^29^ investigated the influence of salinity increase on nitrifying biofilms and reported changes in the microbial diversity, which negatively and weakly influenced the nitrification process. Despite the slight increase in NO_2_^_^–N within the saline treatment groups, no fish mortalities were noted. Moreover, the concentration of NO_3_^_^ was within the desirable range (0–1000 mg/L) for freshwater species^28^, indicating our sandponics system’s functional efficiency. Furthermore, low concentrations of NO_3_^_^ and NH_4_^+^ are attributed to the efficient nitrification process and the root uptake of these forms of nitrogen by plants. Data on fish growth performance metrics and feed utilization indicated that tilapia can be cultured in a sandponics system under different salinities (3000, 6000, and 9000 ppm) with no significant detrimental effects on its growth performance (body weight gain and specific growth rate), feed utilization (feed conversion ratio) and survival. The salinity tolerance of tilapia depends on several factors, such as species, strain, size, and adaptation time, among others^30^. For example, *Oreochromis niloticus* can still survive at water salinities 0–10,000 ppm and dies at salinities above 20,000 ppm, whereas *Oreochromis mossambicus* and *Oreochromis aureus* are highly tolerant and can survive at water salinities exceeding 35,000 ppm^31–33^. The water salinities used in our experiment were moderate for the survival of the fish species (*O. niloticus*) used in this study and thus in agreement with previous studies^26,34,35^. Likewise, the recorded values for fish body weight gain (BWG) in our study were higher than those reported in previous studies^26,34–36^. This could be attributed to differences in the fish’s initial body weight, the study’s duration and experimental fish water conditions.

Soil pH is one of the most important soil properties that directly and indirectly influence plant growth. The ideal pH required for proper plant growth and development ranges between 5.5 and 6.5^37^, depending on the plant species and variety. An increase in soil salinity increases the soil pH depending on the concentration of Na^+^ in the root zone^38^. As such, soil pH values in the current study ranged between 8.5 and 8.6 in all the salinity treatments, including the control. However, the observed soil pH did not cause any detrimental effect on plant growth, thus indicating that kale (Winterbor variety) can tolerate soil pH values above 6.5. It is worth noting that soil pH variations below or above the ideal requirement of the plant species or variety will hinder plant growth due to the unavailability of certain elements for plant-root uptake^37,39^. For instance, trace elements such as Cu, Zn, Fe, and Mn are unavailable for plant uptake at low soil pH^39^. Likewise, an increase in Na^+^ concentration in the root zone influences the bioavailability of certain cations due to the preference of Na^+^ adsorption on negatively charged soil particles. In this study, the soil Fe and Cu composition were lower in highly saline treatments (6000 and 9000 ppm) compared to the control thus indicating displacement of these cations in preference to Na^+^. Moreover, the soil EC increased with increasing salinity as indicated by higher EC values in the 9000 ppm treatment relative to other treatments. An increase in salinity leads to the accumulation of salts in the root zone, which increases the EC^40^.

Kale (*Brassica oleracea*) has the ability to tolerate salinity stress, and certain cultivars, such as *B. oleracea var. acephala* can be grown under salinities reaching up to 230–550 mM sodium chloride (NaCl) with re-watering^41,42^. In this study, kale could tolerate irrigation water salinities reaching up to 6000 ppm without severe detrimental effects on its growth and yield with cuts 1 and 3 recording a higher herbage yield compared to cut 2. The built-up tolerance of kale to salinity stress is attributed to several factors such as increased accumulation of antioxidants, macro and micro mineral elements and osmoprotectants. Vitamin C is a powerful antioxidant and its accumulation in plant tissues is a defensive mechanism that triggers abiotic stress tolerance in plants via non-enzyme scavenging reactions of hydrogen peroxide and reactive oxygen species^43–45^. In the current study, the concentration of vitamin C increased in plant leaf tissues irrigated with saline water, indicating its role in building up the tolerance of plants towards abiotic stress.

In the same light, the increase in potassium and phosphorus contents in leaf tissues could have contributed to the enhanced salinity tolerance of crops under our experimental conditions. This is because previous studies on foliar application of potassium and phosphorous in several plant species, such as *Glycine max* (L.), *Solanum lycopersicum* (L.), and *Phaseolus vulgaris* (L.) cultivated under saline conditions, have indicated improved plant growth and tolerance of crops to salinity stress^46–50^. Moreover, salt-tolerant crops tend to accumulate potassium ions (K^+^) in plant tissues under salinity stress as a defensive mechanism aimed at flashing out the toxic Na^+^ ions from plant tissues^51,52^. Our study also showed an increase in the concentration of certain micro-elements (i.e. copper [Cu]) in leaf tissues of plants irrigated with saline water. Cu has been previously reported to induce antioxidant enzyme activity of certain plant species, such as *Zea mays* and *Solanum lycopersicum* (L.) cultivated under abiotic stress conditions^53–55^. For example, Iqbal et al.^53^ demonstrated that exogenous application of Cu under saline conditions enhanced the antioxidant enzymatic activity and accumulation of osmoprotectants such as proline, sugars, and glycine betaine in corn (*Zea mays*) which improved the crop’s tolerance to salt stress. Likewise, Pérez-Labrada et al.^54^ reported that foliar application of 250 mg/L Cu nano-particles improved the antioxidant activity of catalase (CAT), superoxide dismutase (SOD), ascorbate peroxidase (APX), glutathione (GSH), and glutathione peroxidase (GPX) in leaf tissues of tomato *Solanum lycopersicum* (L.) which in turn improved the plants’ tolerance to salinity stress.

The accumulation of osmoprotectants such as proline and sugars in plant tissues is also another defensive mechanism of plants against osmotic stress and ionic toxicity caused by saline conditions^56^. Osmotic stress hinders the absorption of mineral elements from the growth media hence resulting in retarded plant growth and nutrient deficiency^57^. This study showed that under our experimental conditions, kale can tolerate both osmotic stress and ionic toxicity via the accumulation of proline and sugars in leaf tissues. Previous studies on either accumulation or exogenous application of proline and sugars in plants have indicated improved growth and tolerance of several plant species cultivated under saline conditions^58–62^.

## Conclusion

The present study demonstrated that kale (*Brassica oleracea* L.) grown under integrated aqua-vegeculture systems could tolerate water salinities of up to 6000 ppm without severely compromising the growth, yield, and nutrient composition of the plants. Moreover, channeling fish effluents (organic fertilizer) to the sand grow beds can support the growth of kale under our experimental conditions without any requirements for supplemental fertilization. Rearing Nile tilapia (*Oreochromis niloticus*) in our sandponics systems did not negatively impact the water quality (i.e., dissolved oxygen, ammonia–nitrogen, and nitrate-nitrogen), thus indicating efficient nitrification processes of the system. Likewise, rearing *O. niloticus* at different water salinities (3000 ppm, 6000 ppm, and 9000 ppm) did not negatively impact the growth performance, survival, and feed utilization of fish. Overall, cultivating kale and *O. niloticus* in IAVS in water salinities reaching up to 6000 ppm is a sustainable approach to maximizing food and water production in regions of freshwater scarcity.

## Data Availability

The datasets generated during and/or analyzed during the current study are available from the corresponding author on reasonable request.
